# The in-practice prevention programme: an example of flexible commissioning from Yorkshire and the Humber

**DOI:** 10.1038/s41415-022-4140-y

**Published:** 2022-04-05

**Authors:** Fiona Sandom, Simon Hearnshaw, Siobhan Grant, Lynne Williams, Paul Brocklehurst

**Affiliations:** 41415106774001 School of Health Sciences, Bangor University, UK; 41415106774002 NHS England, North Yorkshire and the Humber Area, UK; 41415106774003 Public Health England, North Yorkshire and the Humber Area, UK

## Abstract

**Introduction ** An In-Practice Prevention (IPP) programme was developed by the Local Dental Network in the North Yorkshire and the Humber area in England in response to an oral health needs assessment. The underpinning logic model drew on a flexible commissioning approach and aimed to incentivise dental teams with NHS contracts to promote the delivery of prevention. This used care pathways that involved the whole dental team and was cost-neutral.

**Aim** The programme was evaluated using realist methodology to identify 'what works, in which circumstances, how and for who?'.

**Design** Realist evaluations are explanatory in nature and attempt to understand the factors that appear to influence the success (or not) of an intervention, rather than demonstrating causality.

**Methods and results ** Following a review of the pertinent literature, semi-structured interviews and focus groups, five theory areas were considered to be critical to the delivery of IPP. In order of stated priority, these were: 1) clinical leadership; 2) 'skill mix'; 3) financial incentives; 4) institutional logic/practice culture; and 5) behaviour change.

**Conclusion** The results appear to show that clinically-led programmes could offer value to dental commissioners within a flexible commissioning model, although this would need to be further tested using an experiment design.

## Key points

- A cost-neutral preventive programme developed by a Local Dental Network in England was explored using realist methodology to understand how it 'worked' in practice.
- Multiple interviews were held iteratively with key stakeholders including dental commissioners, members of the Local Dental Network, general dental practitioners and dental care professionals.
- Clinically-led programmes appear to offer value to dental commissioners, improving both programme adoption and spread while driving innovation and 'peer-to-peer' improvement.

## Introduction

Young children presenting with pain and sepsis resulting from dental caries are difficult patients to manage in high street NHS dental practices in the UK. The experience can also be distressing for young children and their families.^1^^,^^2^^,^^3^ Poor dental experiences at an early age can lead to lifelong dental anxiety and poor patterns of attendance, leading to further health and cost consequences.^4^ The experience of dental caries in young children can have an impact on their quality of life and 'makes a very significant difference to the psychological and social aspects of the child's life'.^5^ It also affects young children's' attendance at school and their educational achievement.^6^^,^^7^^,^^8^ In addition, once the disease is expressed in young children, further dental caries is highly likely.^9^^,^^10^ In 2015, the *North Yorkshire and the Humber oral health needs assessment* in England identified high levels of dental disease amongst young children across deprived communities in the region.^11^ The prevalence of tooth decay in five-year-old children in North Yorkshire and Hull was significantly higher than the England average (43.8% and 43.4% versus 27.9%, respectively). Equally, the severity of tooth decay in five-year-old children in Hull was the third worst in England in terms of the decayed, missing, and filled permanent teeth index (3.78 versus 3.38, respectively).

In response to this, the Local Dental Network (LDN) developed the In-Practice Prevention programme (IPP), which required NHS practices with NHS contracts to identify children (aged between 3-16 years of age) with experience of dental caries (at least one lesion) or those children that required a general anaesthetic. These children were then internally referred to Dental Care Professional (DCP)-led prevention clinics at the practice, where evidence-based prevention (based on *Delivering better oral health*)^12^was delivered over a defined number of appointments, with prescribed evidence-based interventions and oral health messages. Each intervention was associated with a payment (£36 for 3 prevention appointments) and this was offset against the target number of units of dental activity within the Annual Contract Value (ACV). In England and Wales, units of dental activity refer to a banded retrospective payment system which are produced by different types of clinical activity and are capped on an annual basis. This offset was approximately 3% on average, meaning that the prevention activity was resourced within the existing budget (ACV). In this way, the programme aimed to take a flexible approach to local commissioning and provide an incentivised and comprehensive programme to deliver consistent oral health advice and interventions that were targeted at children with the highest levels of disease.

**Fig. 1:**
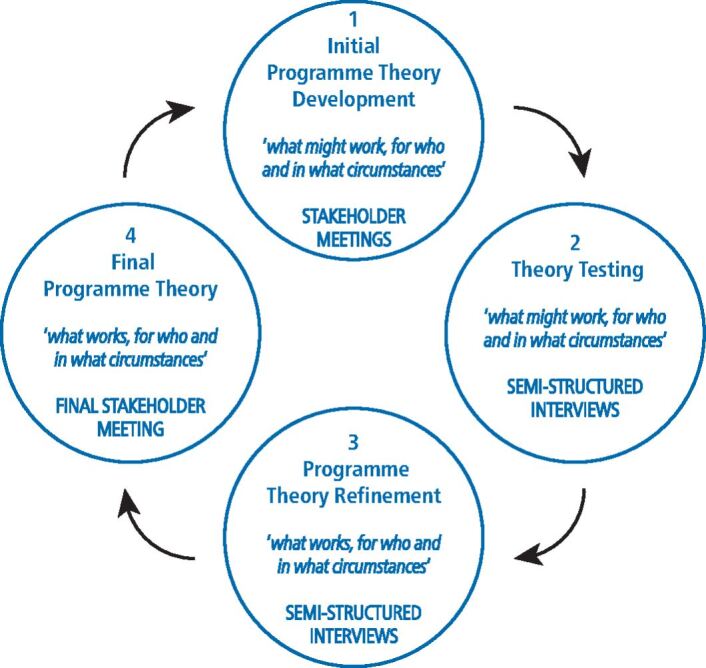
Realist cycle

Realist methodology is a form of theory-driven evaluation and seeks to address the following question: 'what works, for who, how and in what circumstances?'^13^ Realist evaluations are explanatory in nature and attempt to understand the factors that appear to influence the success (or not) of an intervention, rather than demonstrating causality. They are cyclical in nature (Fig. 1) and start by developing a number of statements called initial programme theories (IPTs) that bring together interdependent contexts, mechanisms and outcomes (stage 1).^14^ These are then refined through an iterative process, drawing on evidence from the literature and key stakeholders (stage 2), before refining the programme theory (stage 3) and reviewing the findings with key stakeholders in a final focus group (stage 4). This structured approach produces a rich account and is ratified by key stakeholders at the end of the process. The use of realist methodology is current and popular in health services research as it recognises the need to show how and why interventions work or not, as opposed to merely evaluating whether they work or not.^15^ The aim of this study was to undertake a realist evaluation to understand the experience of stakeholders participating in the IPP programme. This involved following the defined sequence outlined in Figure 1 in order to develop and refine the IPTs using semi-structured interviews.

## Methods

The study was approved on 10 April 2017 (17/LO/0223 Harrow Research Ethics Committee, London). As a 'theory-driven' approach, candidate IPTs were developed *a priori* (stage 1) before iteratively developing these theories using a systematic pattern of enquiry.^16^ This is the standard approach for realist evaluations and is the first stage of the process. It seeks to develop possible explanations for how the IPP programme may work, based on a review of the scientific and grey literature. IPTs are statements that contain at least one context, mechanism and an outcome. In this sense, context describes the socioeconomic, political or organisational environment or background, while an outcome describes what has changed (in behavioural/institutional/organisation/health terms) as a result of the action of a given mechanism. An example of an IPT statement for illustrative purposes is the following: in NHS dentistry (context), setting an ACV (mechanism) contains NHS costs for dentistry at a societal level (outcome). This statement describes one element of the current contract and as can be seen, is relatively simplistic and is a description at a high level (but it is the first step). The role of realist evaluations is to develop a series of these types of statements (stage 1) and then 'unpack' them (stage 2 and 3), so that the researcher gets a detailed understanding of how the intervention in question might work (or not) at a practical level. These are then fed back to a stakeholder group for further refinement to ensure that it captures their experience of the intervention (stage 4).

For stage 1 of the IPP programme evaluation, the following process was followed to ensure that the research team could generate as many potential IPTs as possible:

1. Scoping the scientific and grey literature
2. Concept mining
3. Conceptualising the IPP programme using soft systems
4. Identification of theory areas
5. Literature search
6. Selection and appraisal of documents
7. Data extraction, analysis and synthesis.

Concept mining is a process of exploring potentially important themes that could help explain how an intervention might work (or not). As its name suggests, it involves searching the literature for examples of how evidence-based preventive programmes might 'work' in practice. For the IPP programme, we drew on a number of potentially relevant sources.^12^^,^^17^^,^^18^^,^^19^^,^^20^^,^^21^^,^^22^^,^^23^

This was then followed by two workshops with key stakeholders, where the findings from the concept mining were discussed and detailed questions were asked about the nature of the IPP programme. The structure of the workshops were guided by 'soft systems thinking' using the CATWOE mnemonic (customer, actor, transformation, worldview, owner and environment).^24^ This has been used in a number of realist evaluations and attempts to understand how the intervention works by focusing on these six key elements:^25^^,^^26^^,^^27^

- Customers: beneficiaries of IPP
- Actors: detail of roles and function within the programme
- Transformations: changes required to facilitate IPP
- World views: underlying contextual culture
- Ownership: actors required to drive IPP forward
- Environments: factors that influence implementation.

As the search for potential IPTs developed, a detailed search strategy was developed, taking account of the concept mining and 'soft systems thinking' workshops. Reflecting the realist approach, the search strategy was kept as broad as possible and combined primary and purposive searches in order to capture the most relevant evidence to build, support and/or refute the IPTs that were being developed.^25^ Systematic searches were conducted in three electronic databases: Ovid Medline, CINAHL and PsycInfo, using keywords identified through the search development process and 'keywords' adapted for each information source. The primary search was limited to material from 1990-2019. Abstracts were screened and the evidence summarised into data tables. This was then analysed to develop the IPTs, using a process of triangulation to look for emergent patterns in the data.^26^^,^^27^ This ensured that all the possible underlying contexts, mechanisms and outcomes in the IPP programme were captured.

In stage 2 of the realist process, key stakeholders were invited to participate in semi-structured interviews using an interview schedule based on stage 1. Each participant was asked to reflect on whether or not and in what ways the IPTs explained their experience of the IPP programme. Open-ended questions were also included to identify any new IPTs that the research team had not identified. As a result of the COVID-19 pandemic, the semi-structured interviews were undertaken using Microsoft Teams, audio recorded and transcribed verbatim. In practice, the research team would present each statement to the participant and ask whether it made sense and it what ways their experience differed.

In stage 3 of the realist process, the research team used the data from stage 2 to refine the IPTs further, ensuring that the different context, mechanisms and outcomes were grounded in the experience of the different stakeholders. As can be seen from the example above, when IPTs are developed, they can appear relatively abstract and too simplistic. The idea that setting an ACV (mechanism) in NHS dentistry (context) contains NHS costs for dentistry at a societal level (outcome) doesn't really describe how this works in practice, nor does it capture the nuances of the issues involved. However, it is a good starting point to get at the elements that do matter to key stakeholders, as it helps to frame the discussions. As such, realist approaches are iterative and continue to test and refine the IPTs to build a rich picture of the experiences of those involved.^28^ For the IPP programme, this meant developing an understanding from stage 2 of how the different context, mechanisms and outcomes worked at a practical level, that is, the lived experience of the different stakeholders involved. These refined statements are known as context, mechanism and outcome configurations (CMOCs).^17^^,^^18^

In stage 4 of the realist process, the refined CMOCs were taken to a further group of stakeholders to ratify the final programme theories of IPP. Ahead of the meeting, all the participants were provided with the CMOCs in a diagrammatical form (from stage 3), with a brief explanation of the theory areas. At the beginning of the workshop, participants were first asked to prioritise the five theory areas in order of importance. The research team then utilised 'teacher-learner cycles' before asking the stakeholders to comment.^29^^,^^30^ This is a standard approach in realist evaluations, where the researcher teaches back their findings before asking for any reactions and comments from the group. The programme theories were then finalised.

## Results

Over a two-and-a-half-year period, data from the dental commissioners revealed that the IPP programme had delivered over 17,500 evidence-based DCP-led interventions. During this time, data from the NHS Business Services Authority revealed that attendance patterns and the use of fluoride appeared to increase for child patients. Equally, there was no evidence of any negative influence of the programme on recall rates with adult patients and patient charge revenue, two important concerns for dental commissioners.

Table 1 summarises the output from the soft-systems approach. These findings were divided into five theory areas to focus on the realist synthesis. Given the large number of papers identified in the literature search, each area abstract was reviewed, taking into account their fidelity, trustworthiness, value and relevance to the IPP project. This reduced the number of relevant papers to:

**Table 1:** Key elements of the IPP programme organised according to CATWOE

**CATWOE category**	**Justification**
C: beneficiaries of the IPP programme	The key beneficiaries of the IPP programme were young children in the region with high levels of dental caries The NHS dental practices that worked within the 'flexible commissioning' approach
A: roles and functions in IPP	The Local Dental Network (LDN) was considered to be a key driver for IPP, who were seen as the 'movers and shakers' within local professional circles and so had roles as 'clinical leaders' NHS England were responsible for local commissioning and so were pivotal to the success of the programme and the underpinning 'flexible commissioning' approach Public Health England (PHE) leadership was also seen as critical to ensure a dental public health approach was taken to address the problem The LDN and PHE had developed a business case to take to the NHS England, so multi-agency working was seen as key at a strategic level The engagement of General Dental Practitioners (GDPs) and local dental teams was seen as pivotal (and the incentives and leadership skills needed to promote change at a practice level) Given the change to the 'traditional' commissioning model, the IPP programme had 'national eyes' on the project and so an on-going relationship with the Department of Health was key Members of the dental team (dental practice owners, dental care professionals, dental nurses and dental receptionists) were seen as critical to the delivery of IPP
T: changes and adjustments to implement IPP	Multi-agency and cross-sector working were critical IPP was seen to be 'over and above' what GDPs were normally commissioned to provide, so clinical leadership, culture and behaviour change was key (for example, preparedness to change appointment times to facilitate after-school appointments and increase appointment times) Incentives under-pinned the delivery of the programme GDPs and dental teams needed to understand the problem from a public health perspective (that is, widen their frame of reference and become more 'community-facing') The whole practice team had to engage with the programme (and sometimes there was dissonance between practice owners and their teams, who would deliver IPP) Identification of 'movers and shakers' within the professional was important to promote peer-to-peer acceptance of the programme Addressing NHS England's concern about the impact of the programme on patient charge revenue (PCR) was important In turn, this meant re-focusing NHSE's priority on promoting access to services There was a need to focus on evidence-based prevention and health promotion To facilitate the latter, influencing the attitudes of patients and their families was key
W: underlying context for IPP	IPP to be delivered by dental teams while still working to the existing NHS dental contract (which set targets for activity and performance Availability of suitable appointments for the programme would require a change in the mind-set of the practice and dental receptionists Given this, a change in practice culture was considered to be key The LDN were keen to ensure that the programme was delivered to a consistent standard Given the novelty of the 'flexible commissioning' model, there was a need for the LDN to challenge traditional methods of service provision and challenge national priorities (access/PCR) This required NHSE dental commissioners to allow 'top-slicing' to support IPP National programmes ('Starting Well'; 'Dental Check By One') were also starting to be delivered across England, which could be an alternative to the programme or subsumed into it PHE were driven by the local needs of the population and the need to reduce dental caries among young children At a practice level, different members of the dental team held different world-views about their role
O: factors that influence the ownership of IPP	Elements that determined the success of the programme were identified in the transformations section, but the two key factors that were considered to be critical was the top-level 'buy-in' among the different agencies and the clinical leadership to deliver the programme, through the LDN and the local dental teams in the region
E: contextual barriers	Supportive dental practice owners were needed in order to change current working practices DCPs were to run the programme, who had a different 'world view' to their practice owners Education of the DCPs was fundamental to the implementation of IPP and the consistency of its delivery (this included training of dental nurses in the application of fluoride) A number of specific practice-level barriers were articulated (for example, physical surgery space, capacity within the workforce, willingness to problem solve and the headspace to do this, given the confines of the existing NHS dental contract) Funding of training was not guaranteed (achieved initially through the 'claw-back' mechanism following annual reviews of dental contracts) Practice reorganisation was required to promote role-substitution and role-supplementation (greater use of 'skill mix' in the programme) Changes to internal pay structures within the practice to deliver the programme (and the problems caused if other members of the team on the same pay structures were not involved) Geographical location of practices also posed a potential barrier to the training of dental nurses (who also required time away from the practice or their 'own-time') National priorities on improving access and reducing changes to PCR

1. Institution logic (n = 8)
2. Clinical leadership (n = 9)
3. Financial incentives in the NHS dental contract (n = 10)
4. Behaviour change (n = 9)
5. 'Skill mix' (n = 11).

The IPTs that were subsequently developed from this synthesis are detailed in Table 2. The analysis from the semi-structured interviews (n = 11) in stage 2 appeared to support these statements and reflect a 'mid-range' theoretical position, which could explain both the contexts and mechanisms that led to the outcomes seen in IPP at a practical level (for stage 3). Examples of quotes for this stage are provided in the full report, which is available on the IPP website.^31^

**Table 2:** Initial programme theories

**Theory area**	**Explanation**
Institution logic	1. IF the culture within an NHS practice (context) promotes prevention (mechanism) THEN they are more likely to employ staff with the appropriate skills and knowledge and adopt IPP (outcome) 2. IF the culture within an NHS practice (context) was not clear on the messages within IPP (mechanism) THEN the programme would not be delivered consistently (outcome) 3. IF the 'buy-in' to IPP wasn't consistent (mechanism) across the NHS practice (context) THEN the programme would not be adopted uniformly (outcome) 4. IF the practice principal (practice owner) (context) did not 'own' the programme (mechanism) THEN IPP would not be delivered across the practice (outcome)
Clinical leadership	1. IF clinicians (context) are empowered to take on leadership roles (mechanism) THEN they can play a more significant role in how programmes like IPP are developed and delivered (outcome) 2. IF a programme like IPP is developed in partnership (mechanism) with key stakeholders (context) THEN IPP will be better designed and shaped for use in the NHS practice (outcome) 3. IF clinicians (context) adopt leadership roles (mechanism): THEN they can become empowered to shape change to improve local oral health through IPP (outcome) THEN they can facilitate the implementation of IPP among their peers (peer-to-peer influence) (outcome)
Financial incentives in the NHS dental contract	1. IF NHS practices (context) are provided with financial incentives (or reduction in activity targets) (mechanism): THEN they are more likely to adopt and engage with IPP (outcome) THEN they are more likely to change working practices to facilitate the implement IPP (outcome) 2. IF NHS practices (context) are offered a reduction in their ACV or activity targets (mechanism): THEN it can release sufficient resources to deliver IPP (outcome)
Behaviour change	1. IF NHS practices (context) adopt the evidence-based prevention in IPP (mechanism): THEN young children and their families/carers are more likely to adopt healthy behaviours (outcome) THEN young children and their careers are more likely to attend more regularly (outcome) THEN young children are more likely to improve their oral health (outcome)
'Skill mix'	1. IF NHS practices (context) adopt greater levels of 'skill mix' (mechanism): THEN the practice is more likely to promote IPP (outcome) THEN they are more likely to meet future population need (oral health) via programmes like IPP (outcome) THEN it can free dentists to undertake more complex cases (pursuant to their training) (outcome)

**Fig. 2:**
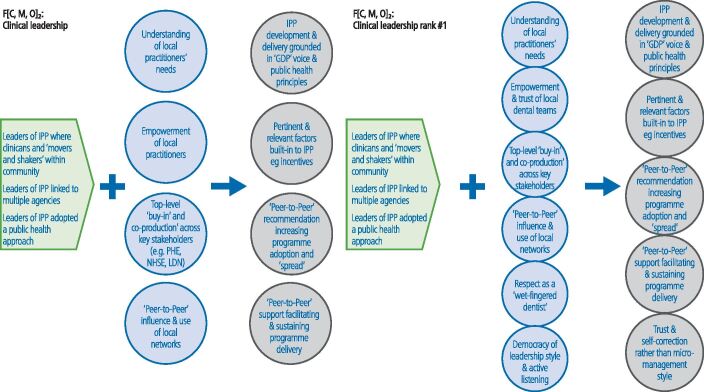
Changes made to the CMOC on clinical leadership (note: context = green; mechanism = blue; outcome = grey)

**Fig. 3:**
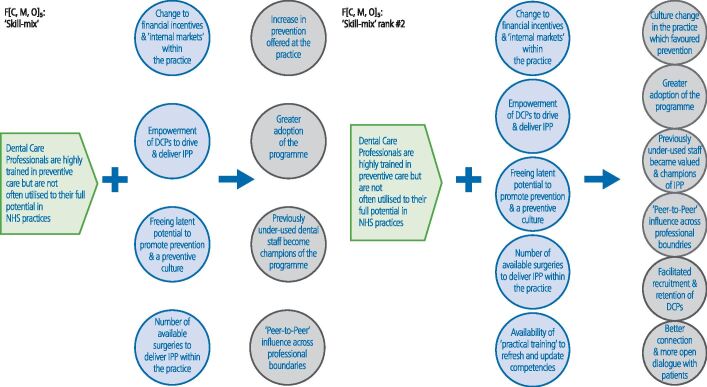
Changes made to the CMOC on 'skill mix' (note: context = green; mechanism = blue; outcome = grey)

**Fig. 4:**
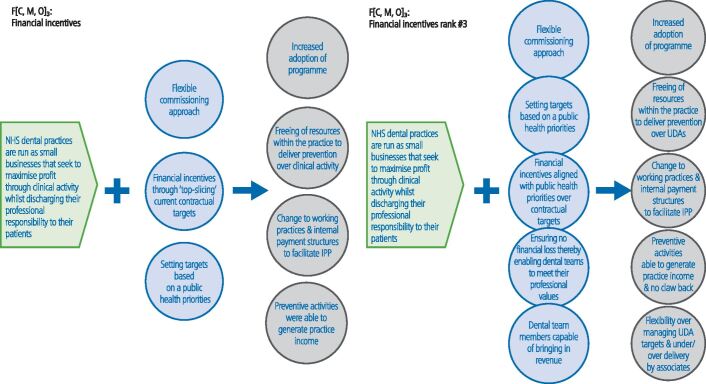
Changes made to the CMOC on financial incentives (note: context = green; mechanism = blue; outcome = grey)

**Fig. 5:**
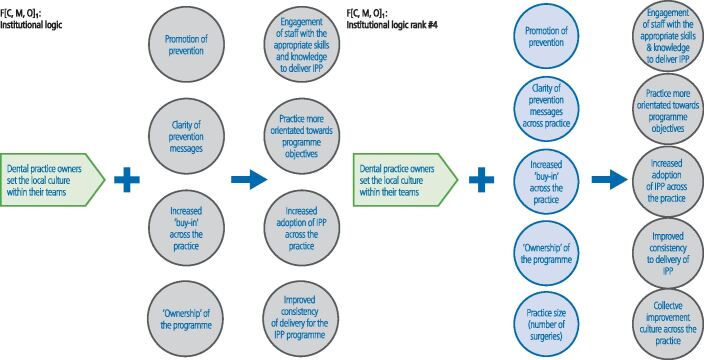
Changes made to the CMOC on institutional logic (note: context = green; mechanism = blue; outcome = grey)

**Fig. 6:**
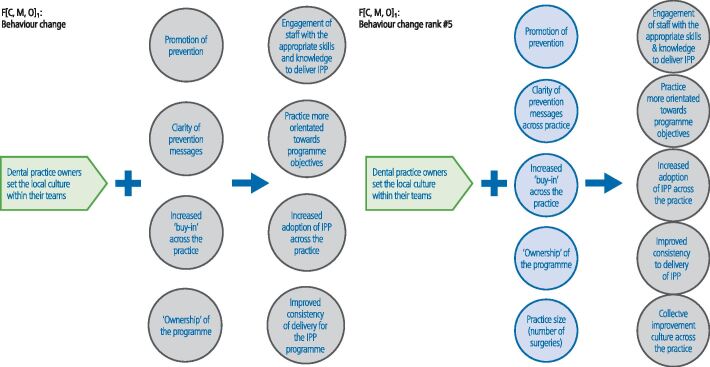
Changes made to the CMOC on behaviour change (note: context = green; mechanism = blue; outcome = grey)

In the final workshop (n = 10), the ranking of the theory areas was as follows: CMOC 1) clinical leadership; CMOC 2) 'skill mix'; CMOC 3) financial incentives; CMOC 4) institutional logic/practice culture; and CMO 5) behaviour change (Figures 2, 3, 4, 5 and 6; context in green, mechanism in blue and outcome in grey). The latter two theory areas were seen by the stakeholders to be dependent on the former three.

A further two mechanisms and an additional outcome were added to CMOC 1 (clinical leadership) before finalising (Fig. 2). The role of 'peer champions' was considered to be key:

- '*It was much more powerful coming from [the practice owner] as the peer champion. So he was able to say to them, I know this is really new and it's a bit scary, but I've walked the walk and it's fine*' (Public Health England [PHE] representative)
- '*The outcome of the peer-to-peer influence and the feedback that resulted, that loop created a responsive programme that allowed the programme to evolve so that strengths were incorporated and any problems could be addressed*' (commissioner).

'Skill mix' (CMOC 2) was considered to be the second most important theory area (Fig. 3). A further mechanism and two outcomes were added to the CMOC. The role of DCPs was seen as pivotal to driving IPP forward in the practice and in communicating with patients:

- '*I think one [most important element] was the DCPs supporting each other and leading each other*' (PHE representative)
- '*but the DCPs did roll it out in the practices, so it was all led by them really [...] they were vital to rolling it out*' (practice owner)
- '*I've seen it with my own eyes, where the patients had a better connection with the DCPs*' (practice owner).

Financial incentives (CMOC 3) were also key (Fig. 4). A further two mechanisms and outcomes were added. Generating additional income was not seen as a prerequisite for success, more that dental practices were released from the necessity of meeting ACV targets:

- '*Providing you're not disadvantaged financially, if you're disadvantaged, then it's a no go. But you don't have to have a huge financial gain, it can be just the status quo as far as finances go, providing if then there are other benefits to doing it and it's more rewarding work*' (practice owner)
- '*It can't be financially disadvantageous, but there are other things that are important in terms of improving the quality of your working day really and the working balance by using a different skill mix*' (PHE representative).

CMOC 4 (institutional logic/practice culture) and CMOC 5 (behaviour change) were also considered to be important (Figures 5 and 6), but as highlighted above, it was argued that they were reliant on the first three theory areas being in place.

## Discussion

The results of the realist evaluation appear to show that clinical leadership, 'skill mix' and financial incentives were important to the success of IPP. A number of clinically-led programmes have been evaluated and have demonstrated the potential of LDNs.^17^^,^^32^ Findings in this study support this and demonstrate the importance of 'clinically owned and clinically led' initiatives (CMOC 1) in driving targeted clinical activity (Fig. 2). In IPP, the programme leaders were respected members of their local dental community and had been practice owners. As a result, they understood the needs of local practitioners and were able to exert 'peer-to-peer' influence, which was then cascaded down and across practice owners, increasing programme adoption and spread. They were also able to ensure that the financial incentives within IPP where appropriate and were aligned with public health objectives.

The use of 'skill mix' in NHS dentistry has often lagged behind that seen in medical specialties.^33^ As highlighted by Barnes *et al*., many of the influences on DCPs are associated with the financial incentives in the contract (CMOC 3) and institutional logics of the NHS dental practice (CMOC 4).^34^ IPP promoted 'skill mix' and for many of those interviewed, it appeared to empower the DCPs involved, particularly the dental nurses. This 'freed up' latent potential within the dental teams to champion and promote a preventive culture to deliver the increase in care (Fig. 2). This phenomenon is also seen in healthcare where nurses act as 'human intermediaries'. This is a term used to describe interpersonal contact to facilitate knowledge exchange through expertise and a 'range of interchangeable roles between producers and users of evidence'.^35^^,^^36^^,^^37^ In IPP, DCPs within the practice were able to exert influence on the actions of their colleagues, guiding them towards evidenced-based approaches to care.^38^^,^^39^^,^^40^

Financial incentives were also key. However, it was argued that this had more to do with reducing the need to chase ACV targets and the release of latent capacity within the practice (using DCPs). This appears to support the importance of 'flexible commissioning', where targets are aligned to local public health objectives. As highlighted above, all of the interviewed practice owners believed this was key to the adoption of IPP, which was cost neutral for commissioners. Equally, this enabled practice owners to change their working practices and some reported that they were able to change their internal payment structures to facilitate IPP without impacting on care for adult patients (Fig. 2).

Goodwin *et al.*, argue that institutional logics within NHS dentistry not only includes dental practices as businesses, but also professional ethics and contextual factors, based on where the practice is embedded.^23^ As such, the drive to maintain (and maximise) the viability of an NHS practice can also be tempered by a practice owner's view about their sense of duty to their patients and their ideas about how best to deliver care for their patients and community. This was supported in the findings of the IPP evaluation.

## Conclusion

Given the findings of the realist evaluation, it would appear that clinical leadership, 'skill mix' and financial incentives were seen as the most important elements of the IPP programme. Aligning public health priorities with potential financial incentives within the existing NHS contract was key. Equally, the utilisation of the whole of the dental team was critical for the success of the IPP programme and created local champions that drove the institutional logic within the practice and behaviour change.
